# Diagnostic applications for Lassa fever in limited-resource settings

**DOI:** 10.1136/bmjgh-2018-001119

**Published:** 2019-02-07

**Authors:** Devy M Emperador, Solomon A Yimer, Laura T Mazzola, Gunnstein Norheim, Cassandra Kelly-Cirino

**Affiliations:** 1 Foundation for Innovative New Diagnostics (FIND), Emerging Threats Programme, Geneva, Switzerland; 2 Department of Vaccine Science, Coalition for Epidemic Preparedness Innovation (CEPI), Oslo, Norway

**Keywords:** lassa fever, lassa virus, LASV, in vitro diagnostics, point of care

## Abstract

Lassa fever, caused by arenavirus Lassa virus (LASV), is an acute viral haemorrhagic disease that affects up to an estimated 300 000 individuals and causes up to 5000 deaths per year in West Africa. Currently available LASV diagnostic methods are difficult to operationalise in low-resource health centres and may be less sensitive to detecting all known or emerging LASV strains. To prioritise diagnostic development for LASV, we assessed the diagnostic applications for case detection, clinical management, surveillance, outbreak response, and therapeutic and vaccine development at various healthcare levels. Diagnostic development should prioritise point-of-care and near-patient diagnostics, especially those with the ability to detect all lineages of LASV, as they would allow for rapid detection in resource-limited health facilities closer to the patient.

**Summary box**

- Diagnostics for Lassa virus (LASV), which causes acute haemorrhagic disease, are essential for case detection and management, ongoing surveillance, outbreak responses, and assessment of vaccine and drug efficacy and effectiveness.
- Existing diagnostic tests are limited in terms of strain coverage and suitability for use in low-resource healthcare settings.
- Target product profiles for LASV diagnostics should take into account the need for point-of-care testing and detection of multiple strains, to encourage the development of new diagnostics or improvement of existing tests that can effectively address this threat to public health.

## Introduction

Despite measures to prevent and control the spread of Lassa virus (LASV), the fight against Lassa fever is hampered by a lack of vaccines, well-documented therapeutics and appropriate diagnostics.^1 2^ There are currently no commercially available diagnostics capable of capturing the diversity among viral strains and providing a diagnosis at any time-point during the clinical course of illness. This indicates that the available LASV diagnostic methods are difficult to operationalise in low-resource health centres and may be less sensitive to detecting new or emerging LASV strains. Building on the Lassa fever diagnostic landscape presented by Mazzola and Kelly-Cirino,^1^ here we present diagnostic applications to help define priority development for the prevention and control of Lassa fever in LASV-affected countries.

## Challenges in LASV detection

The development of novel diagnostics requires thorough understanding of the pathophysiology, pathogenesis and genetic diversity of LASV. The majority of patients who become infected with LASV are asymptomatic or have mild symptoms such as fever, weakness and malaise, which often go unreported.^3 4^ For those who do seek care, these symptoms mimic other endemic diseases like malaria, typhoid fever and other viral haemorrhagic fevers, resulting in misdiagnosis or missed cases of LASV.^5^

The pathogenesis of LASV is marked by its inhibited immune response as LASV infection fails to lead to the activation or maturation of the primary target cells for innate immunity (dendritic cells and macrophages). In general, high viraemia is correlated with LASV disease severity,^3^ with viraemia peaking between 4 and 9 days after disease onset.^6–8^ LASV hinders the development of immune cells that are crucial to immune response and resistance to infection.^9^ The LASV small matrix protein and nucleoprotein block interferon responses by thwarting viral RNA sensing, preventing antigen presentation and downstream T cell responses.^10^ A robust and early T cell response in humans is a critical marker for LASV survival compared with B cell response.^11^ There are, however, limited data on the natural human immune response to LASV, and is a key research gap for countermeasure and diagnostic development.

The high level of genetic diversity between LASVs also poses a diagnostic challenge as only <75% of the genome sequence is conserved. Phylogenetic analysis based on whole genome sequencing showed that LASV is grouped into four main lineages.^1 2^ A fifth ‘AV’ lineage from Mali/Cote D’Ivoire has recently been proposed.^12^ New Lassa diagnostic development initiatives should take into account LASV genetic diversity in their assay/product development process.

Another significant challenge in LASV testing is the high-containment safety requirement for confirmatory testing, as well as the development and validation studies of new diagnostics.^13^ Symptomatic patients in both rural and urban settings are most likely to present at health facilities with limited biosafety capacity (table 1). While testing in the community level (or level 0), whether through community health workers (CHWs) or village health teams (VHTs) outside of the health facility, or self-testing can support disease detection and linkage to care,^14 15^ proper biosafety precautions required for LASV and other viral haemorrhagic fevers may not always be followed, especially if an outbreak is undeclared. The majority of CHWs or VHTs will have minimal training in primary care and refer most ill individuals to the next level health facility.

**Table 1:** Healthcare level and testing capabilities in Lassa-affected countries*

Laboratory facility level	Test users	Country examples	
		Nigeria^30 31^	Sierra Leone^32^
0 Community level	Community members, healthcare workers	Local community	Local community
1 Primary care setting	Healthcare workers	Primary health centre at the village and local level	Primary health centre at the local level
2 District laboratory	Laboratory technicians	Secondary health facilities at the state level	District health centre/hospital
3 Regional, provincial, specialised laboratories	Senior laboratory specialists/technicians	Tertiary health facilities at the federal level, for example, ILFRC at ISTH, Irrua	District health centre/hospital, for example, KGH, Kenema
4 National reference laboratory	Senior laboratory specialists	Nigeria Centre for Disease Control, Abuja	Central Public Health Reference Laboratory, Freetown

*Adapted from the *WHO Consultation on Technical and Operational Recommendations for Clinical Laboratory Testing Harmonization and Standardization*.^17^

ILFRC, Institute of Lassa fever Research and Control; ISTH, Irrua Specialist Teaching Hospital; KGH, Kenema Government Hospital.

For individuals who seek care, level 1 health facilities are limited to offering basic outpatient services and the capacity for simple sample collection and diagnostic testing.^16 17^ Level 2 and level 3 health facilities serve a larger region and care for patients referred from level 1 health facilities and offer more specialised staff and equipment, and often include dedicated laboratory space and trained laboratorians. In low-resource settings, many district and regional hospitals can fall between the level 2 and level 3 health facility distinction.^17^

In contrast, level 4 health facilities serve as a reference health facility for level 3 facilities and below. Level 4 facilities will have highly trained staff and sophisticated equipment, allowing them to run a number of tests that cannot be performed in lower level laboratories, such as sequencing and drug resistance and susceptibility testing. Reference laboratories will have the capacity to test higher biosafety level pathogens, including LASV, but may not be located in a country experiencing a Lassa fever outbreak and are rarely the first point of contact where patients present for care and diagnosis. In this respect, international reference laboratories as organised in the WHO Dangerous Pathogens Laboratory Network are critical for verification, quality control and the specialised analyses affecting countermeasure development.

## Diagnostic applications for Lassa fever

To guide the development of diagnostics for Lassa fever, one must take into account the health facility level where individuals access and receive care, the test users who will conduct and report the test results, and the response algorithm to confirmed Lassa fever cases. Here, point-of-care (POC) and near-patient diagnostics refer to cartridge-based platforms, and are ideal for testing in resource-limited health facilities as they can be set up and used outside of a dedicated laboratory space and are easier to operate. These tests may still require trained laboratorians for sample processing and test conduct. Rapid diagnostic tests (RDTs), which specifically leverage antibody/antigen capture agents similar to an ELISA (eg, lateral flow dipsticks), provide rapid results (10–30 min) and can be used by individuals with minimal training. Below, we describe different diagnostic use cases for LASV, with attention spent on the most appropriate diagnostic for case detection and clinical management, surveillance, outbreak response, and therapeutic and vaccine development (table 2).

**Table 2:** Use cases for LASV diagnostics: needs based on target use and setting in a Lassa-affected country

Intended use case	Target use setting/diagnostic needs				
	4	3	2	1	0
	National reference lab	Referral/regional hospital	District hospitals	Health centres	Health posts, field settings
Case detection and management	MDx*† POC MDx*† IA*†	MDx*† POC MDx*† IA*	POC MDx* POC IA* RDT*		
Surveillance	MDx*† sequencing IA*	MDx* POC MDx* IA*	POC MDx* POC IA* RDT*	RDT*	
Outbreak response	MDx*† POC MDx*† sequencing IA*	MDx* POC MDx* IA*	POC MDx^*^ POC IA* RDT*	RDT*	RDT*
Clinical trials (vaccine, therapeutics)	MDx*† POC MDx*† sequencing IA*†	MDx*† POC MDx*† IA*†	POC MDx* POC IA*		

*Requires pan-LASV detection.

†Requires pan-VHF differentiation.

IA, immunoassay (eg, ELISA); LASV, Lassa virus; MDx, molecular diagnostics (eg, reverse transcriptase PCR); POC, point-of-care or near-patient; RDT, rapid diagnostic test (eg, lateral flow immunoassay); VHF, Viral haemorrhagic fever.

### Case detection and management

Early diagnosis of LASV infections remains a challenge despite the knowledge of Lassa fever epidemiology and symptoms by healthcare providers in LASV-affected regions^18^ due to the non-specific nature of many of the common symptoms associated with Lassa fever. As febrile patients present to a health facility and LASV infection may not be suspected, diagnostics for clinical management should be able to accurately detect LASV infection.

Febrile individuals in LASV-affected regions will present at their local health centre, most likely at a primary healthcare facility (eg, level 1 or 2 health facilities). The ideal diagnostic would include simple set-up and sample collection, rapid and easy-to-read results, the ability to withstand field transport and use, high sensitivity, and the ability to detect multiple LASV lineages. Due to limited resources and potential biosafety concerns among healthcare workers, POC or near-patient molecular diagnostics (MDx) and immunoassay (IA) platforms would be most useful to detect LASV clade-specific and LASV-specific antigen, respectively, allowing for sample preparation and testing in a contained unit. For level 1 and 2 health facilities with minimal equipment, an RDT that can detect LASV antigen may be more beneficial. These tests should be able to detect LASV independently of the multiple LASV lineages, especially those that circulate in the region, and differentiate between LASV and other fever-causing agents to properly rule out other causes of febrile illness. Results from these tests can be qualitative; however, a quantitative read-out of viral load may be useful to assess disease severity and to support clinical management.^3 6^

Confirmatory testing will most likely be required for LASV-positive results from lower level health facilities; these tests will be done at a level 3 or 4 health facility with biocontainment capabilities. In these laboratory settings, the ideal diagnostic would provide rapid quantitative results, have high sensitivity, and have the ability to detect and distinguish multiple LASV antigens. In the level 3 health facility, a POC or near-patient MDx or a POC IA that can detect LASV antigen or LASV-specific antibodies (IgM or IgG) would also be ideal. Sequencing can be completed in level 4 reference laboratories, although time-consuming and improvements to primers may be required to ensure more complete strain coverage and detection sensitivity. International reference laboratories and national biopreparedness laboratories outside the affected countries will have use cases in the same range as level 4 laboratories but more capabilities in terms of viral culture and sequencing.

### Surveillance

Surveillance of circulating LASV in endemic countries provides adequate knowledge that can be used for diagnosis and treatment of patients and ultimate reduction of the overall disease burden. In addition, well-documented prevalence and incidence of LASV infection are crucial for the design and execution of clinical trials for therapeutic and vaccine development to harmonise a case definition that can be used as an endpoint. To date, current surveillance data for LASV are limited in the majority of LASV-affected countries due to the lack of readily accessible and deployable diagnostics.

As LASV-infected individuals with more severe disease present to a health centre,^19^ results from diagnostic tests in level 1 and 2 facilities should be connected to a centralised, national surveillance system to track the incidence of LASV infection. Similarly, an IA that can detect LASV-specific IgG antibodies may be useful for seroprevalence studies, which are needed to assist with clinical trial design. Sequencing can also be useful to understand the epidemiology of circulating LASV strains in LASV-affected countries and can be used to assist with vaccine efficacy and effectiveness studies, although level 4 reference laboratories will likely be the only labs capable of conducting routine sequencing.^20^

### Outbreak response

Once an outbreak of Lassa fever has been identified, all levels of the healthcare continuum will be engaged in response. In general, diagnostics for LASV outbreak response should be able to rapidly diagnose and/or confirm LASV infection to aid in proper case detection and triage, although the ideal diagnostic will differ based on where patients enter care.

The goal in level 1 and 2 health facilities is to rapidly detect suspected cases and to confirm LASV infection for patient triage and care. Similar to the diagnostics recommended for case detection and clinical management, a POC or near-patient MDx or IA that can rapidly detect LASV, preferably for multiple LASV lineages, would be ideal. A quantitative or semiquantitative MDx test would also be useful in order to assess disease severity and assist with patient triage in the health facility. Likewise, an RDT that can detect LASV antigen would be useful for screening and triage in the health facility, but will need to have high sensitivity or be part of a test algorithm with a confirmatory test to confirm LASV infection.

For confirmatory testing in level 3 and 4 health facilities, the diagnostic goal is to rapidly detect LASV infection and to confirm LASV infection for samples referred from lower level health facilities. In level 3 health facilities, useful diagnostics include a POC or near-patient MDx that can rapidly detect LASV and a POC IA specific to LASV antigen for confirmatory testing. At a level 4 reference laboratory, the ideal diagnostic would provide rapid quantitative results, high sensitivity, and the ability to detect and distinguish multiple LASV lineages. A benchtop MDx and IA would be easily deployable in this setting, although improvements to reagents would be required to ensure more complete strain coverage and detection sensitivity.

As confirmed patients are isolated and entered into care, response teams will quickly deploy into the community for contact tracing. In the community, the diagnostic goal is to rapidly detect suspected cases to prevent the further spread of LASV and quickly enter individuals into care. In this setting, the diagnostics must be simple to set up, have easy sample collection, provide rapid and easy-to-read qualitative test results, have the ability to withstand field transport and use, be highly sensitive, and have the ability to detect multiple LASV lineages. An RDT that can detect LASV antigen would be particularly useful for contact tracing and case detection in the field, although confirmatory testing will be required for suspected LASV cases.

### Therapeutic and vaccine development

Currently there are no effective therapeutics and vaccines commercially available to treat and prevent Lassa fever. However, with products in the pipeline for clinical evaluation, an understanding of the types and regulatory requirements for tests employed in case verification for efficacy trials is needed. The laboratory tests needed for therapeutic development depend on the stage and nature of the therapeutic candidate (eg, chemical compounds or biologics, which include monoclonal antibodies). Similarly, the types of laboratory tests needed for vaccine development depends on the stages (phases) and nature of the vaccine candidates. While we acknowledge the importance of preclinical laboratory studies to establish the therapeutic efficacy of a compound in animal models,^21^ we will only discuss laboratory diagnostics required in human clinical trials.

#### Phase I /II

Phase I trials are conducted to define acceptable safety and reactogenicity of a therapeutic or vaccine candidate, as well as to determine dose ranges for the final product.^22^ For vaccine candidates, the phase I trial also assesses the immunogenicity of the candidate. Regardless of the product type, phase I studies are non-randomised, small-scale studies normally comprising <100 healthy volunteers. The majority of phase I trials are conducted in high-resource settings with the capacity to perform advanced laboratory techniques for immunological, genomics, proteomics and transcriptional analyses.

Generally, the diagnostics needed to assess the therapeutic or vaccine candidate include highly sensitive and specific tests to detect LASV infection among individuals who receive the therapeutic or vaccine versus placebo or control. Specific to vaccine trials, tests that assess the immune response to the vaccine are required and may vary depending on the vaccine platform and correlate(s) of protection to LASV infection (table 3).

**Table 3:** Examples of the role of diagnostics in assessing vaccine immune responses^9^

Test type	Examples
Antibody tests	ELISA-binding assay: to detect LASV-binding antibodies and cross-reaction between clades (clades I–IV). ELISA-Competition: to define epitope specificity of binding antibodies to LASV GP. Neutralisation assay-LASV GPC: to detect antiviral neutralising antibodies/breadth of response (clades I–IV).
T cell response tests	ELISPOT: to quantify LASV vaccine-induced specific T cell responses or to quantify vector-specific T cell responses. Vectored antigen presentation assay.
Immune profiling	LASV-specific CD4 and CD8 T cells: to perform phenotypic and functional characterisation of T cells; to characterise vector-specific T cell response. Transcriptomics: gene expression profile characterisation of early immune responses and to identify potential effector function correlates.
Antigen tests	ELISA assay: to detect LASV viral protein. RDT: to detect LASV viral protein.
Nucleic acid tests	qRT-PCR: detection of vector RNA.

ELISPOT, enzyme-linked immunospot; GP, glycoprotein; GPC, glycoprotein precursor; LASV, Lassa virus; qRT-PCR, quantitative reverse transcriptase PCR; RDT, rapid diagnostic test.

The major difference between phase I and phase II studies is that phase II studies involve larger numbers of participants, are randomised and include controls. The objective of the phase II trial is to identify the vaccine/drug preparation and optimal dose, and to determine the appropriate vaccination schedule to be considered for phase III trials. Phase II trials provide preliminary information on the protective efficacy of the relevant active component(s) in either reducing morbidity or preventing disease and on the safety profile of the candidate therapeutic or vaccine.^22 23^

#### Phase III

Phase III trials are large-scale, randomised controlled clinical trials enrolling between 300 and thousands of subjects from a heterogeneous target population. Phase III trials mainly aim at determining the clinical efficacy and safety of a specific vaccine/drug product. A phase III trial is essential to pave the way for registration and licensure approval of a vaccine/drug formulation.^23^ Phase IIb and phase III trials will most likely be conducted in selected Lassa-affected countries, in health centres with the capacity to run the clinical trials. With prior capacity building, level 2–4 health facilities will be the most likely locations for clinical trial testing. In more well-resourced laboratories (eg, level 3–4 health facilities), the diagnostics used to assess safety and efficacy during the phase I trial can be used, especially those for assessing correlates of protection and LASV infection. In less resourced laboratories (eg, level 2 health facilities), a POC MDx or POC IA would be ideal for assessing LASV correlates of protection and infection. In each location, the diagnostic used must be able to detect multiple LASV lineages to confirm LASV among trial participants, as well as to provide proper case management for participants with non-LASV disease. Importantly, diagnostics that can reliably distinguish between active infection and the effects of vaccination will be critical in understanding vaccine efficacy in these trials, potentially requiring a combination of molecular and serological testing to confirm protection.

Beyond the clinical trials, diagnostics will be needed to ensure monitoring of the effect during roll-out and delivery of an LASV vaccine or therapeutic. Similar to the diagnostics used in the phase III trials, diagnostics in the post-LASV vaccine era should also be able to distinguish between acute infection and vaccine protection.

## Conclusion

Based on the diagnostic applications described and with a focus on patient outcomes, POC and near-patient diagnostics should be prioritised for development. POC and near-patient diagnostics, especially molecular-based, can be used at multiple health facility levels to diagnose and confirm patients with suspected LASV infection for outbreak response, clinical management, routine surveillance and clinical trials. Despite LASV strains remaining regionally located, POC diagnostics should be able to detect multiple LASV circulating strains to capture introductions of LASV strains in different countries; this may incentivise diagnostic developers to invest in LASV testing because of the use of these diagnostics in multiple LASV-affected regions. Similarly, as the need for syndromic panels has been highlighted,^24^ LASV POC diagnostics should be able to differentiate between LASV and other pathogens to improve fever management in regions with other cocirculating pathogens that cause fever.

Beyond LASV testing, the development of POC and near-patient test platforms for use in these settings must allow for expanded testing of other pathogens or clinical biomarkers. This would result in laboratory strengthening for routine testing of other pathogens (eg, HIV, tuberculosis, malaria) in times of low LASV circulation. Given the recent release of the WHO’s Essential Diagnostics List, laboratories would also benefit from integrated platforms that can assist with the detection and management of non-communicable diseases.^25 26^ Allowing for multiple uses of a POC test platform also incentivises product development as a larger market use will be attractive to developers and can result in more affordable products for purchase.

Lassa fever RDTs, which are generally less expensive compared with other diagnostic platforms, are useful to screen and detect patients with suspected LASV in low-resource health facilities or in the community, although the performance of the current assays prevents their widespread use. Efforts into improving assay sensitivity and lineage coverage are needed to promote their use during an outbreak.

Besides the need for diagnostics, other gaps remain in the detection and management of Lassa fever. There is a need to strengthen laboratory capacity in LASV-affected countries in order to implement both currently available tests as well as newly developed diagnostics. There may also be a need to harmonise LASV case definitions in affected countries. Following case verification, which will be strengthened by new diagnostics, effective and safe therapeutics and vaccines are required to ensure the treatment and prevention of LASV infection in the long term. Encouragingly, there are significant gains in the assessment of antivirals for treatment and international efforts in supporting vaccine development for Lassa fever.^27–29^ For example, an initiative to address the rapid development of new vaccines is the Coalition for Epidemic Preparedness Innovations, which based on WHO’s priority disease list will fund up to six vaccine candidates to enter clinical phase I trials in 2019–2020 and reach phase II by 2021.

Lassa fever continues to pose a public health threat in West Africa. Diagnostics are key in the early detection of, as well as rapid response to, Lassa fever outbreaks, and important to assess vaccine and drug efficacy and effectiveness. While there are tests available for the detection of LASV, commercially available diagnostics that are affordable, for use near the patient and suitable for testing in limited-resource settings are needed. To support the goals of the WHO R&D Blueprint Initiative for Lassa fever, target product profiles for LASV diagnostics should take into account the need for POC or near-patient tests to spur the development of new diagnostics or improvement of currently available tests.
